# Formation of Flavonoid Metabolons: Functional Significance of Protein-Protein Interactions and Impact on Flavonoid Chemodiversity

**DOI:** 10.3389/fpls.2019.00821

**Published:** 2019-07-09

**Authors:** Toru Nakayama, Seiji Takahashi, Toshiyuki Waki

**Affiliations:** Department of Biomolecular Engineering, Graduate School of Engineering, Tohoku University, Sendai, Japan

**Keywords:** metabolon, flavonoids, chemodiversity, biosynthesis, protein-protein interaction, binary interaction, cytochrome P450, ER

## Abstract

Flavonoids are a class of plant specialized metabolites with more than 6,900 known structures and play important roles in plant survival and reproduction. These metabolites are derived from *p*-coumaroyl-CoA *via* the sequential actions of a variety of flavonoid enzymes, which have been proposed to form weakly bound, ordered protein complexes termed flavonoid metabolons. This review discusses the impacts of the formation of flavonoid metabolons on the chemodiversity of flavonoids. Specific protein-protein interactions in the metabolons of *Arabidopsis thaliana* and other plant species have been studied for two decades. In many cases, metabolons are associated with the ER membrane, with ER-bound cytochromes P450 hypothesized to serve as nuclei for metabolon formation. Indeed, cytochromes P450 have been found to be components of flavonoid metabolons in rice, snapdragon, torenia, and soybean. Recent studies illustrate the importance of specific interactions for the efficient production and temporal/spatial distribution of flavonoids. For example, in diverse plant species, catalytically inactive type-IV chalcone isomerase-like protein serves as an enhancer of flavonoid production *via* its involvement in flavonoid metabolons. In soybean roots, a specific isozyme of chalcone reductase (CHR) interacts with 2-hydroxyisoflavanone synthase, to which chalcone synthase (CHS) can also bind, providing a mechanism to prevent the loss of the unstable CHR substrate during its transfer from CHS to CHR. Thus, diversification in chemical structures and temporal/spatial distribution patterns of flavonoids in plants is likely to be mediated by the formation of specific flavonoid metabolons *via* specific protein-protein interactions.

## Introduction

**Figure 1:**
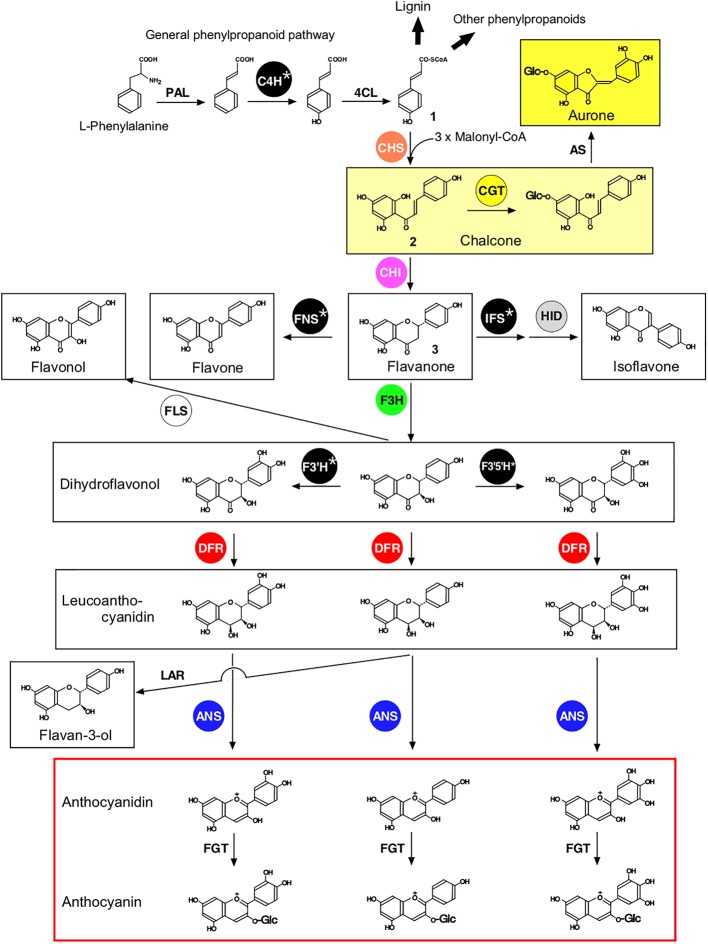
Proposed general pathways of flavonoid biosynthesis. Ten flavonoid classes are shown within boxes. Enzymes that are discussed in terms of their protein-protein interactions in this review are shown within circles. Enzyme abbreviations are: PAL, phenylalanine ammonia lyase; C4H‚ cinnamate 4-hydroxylase; 4CL‚ 4-coumarate:CoA ligase; CHS, chalcone synthase; CGT, chalcone 4′-*O*-glucosyltransferase; AS, aureusidin synthase; IFS, 2-hydroxyisoflavanone synthase; HID, 2-hydroxyisoflavanone dehydratase; CHI‚ chalcone isomerase; FNS, flavone synthase; F3H, flavanone 3-hydroxylase; FLS, flavonol synthase; F3′H, flavonoid 3′-hydroxylase; F3′5′H, flavonoid 3′,5′-hydroxylase; DFR‚ dihydroflavonol 4-reductase; ANS, anthocyanidin synthase; LAR, leucoanthocyanidin 4-reductase; FGT‚ flavonoid 3-*O*-glucosyltransferase. Note that F3′H and F3′5′H may act on flavanones, flavones, and flavonols, depending on the plant species (not shown). Flavonoids and related metabolites are *p*-coumaroyl-CoA (**1**), 2′,4,4′,6′-tetrahydroxychalcone (THC) (**2**), and naringenin (**3**).

Flavonoids are a class of plant specialized metabolites with a basic C6-C3-C6 skeleton, for which 10 major classes (i.e., chalcones, aurones, flavanones, flavones, isoflavones, dihydroflavonols, flavonols, leucoanthocyanidins, anthocyanidins, and flavan-3-ols) have been described (Figure 1). In nature, flavonoids generally occur as glycosides or acylglycosides, with more than 6,900 different structures (Arita and Suwa, 2008). Each plant lineage produces structurally distinct flavonoids in a lineage-specific manner, which play important roles in plant survival and reproduction. For example, in many cases, flower colors arise from anthocyanins and other flavonoids, which contribute to attracting pollinators (Andersen and Markham, 2006). In legumes, (iso)flavonoids in root exudates serve as chemoattractants for specific symbiotic bacteria as well as genetic inducers of nodulation (Barz and Welle, 1992; Subramanian et al., 2006, 2007). These (iso)flavonoids also play important roles in plant defensive mechanisms against infections by pathogens and invasion by herbivores (Aoki et al., 2000). Moreover, consumption of flavonoids is relevant for human nutrition, as illustrated by soybean [*Glycine max* (L.) Merr.] isoflavones, which exhibit estrogen-like and antioxidant activities and have been implicated in the ability of soy to prevent hormone-dependent cancers and cardiovascular diseases (Wiseman, 2006). These diverse bioactivities of flavonoids in plant biology and human nutrition are closely related to their diversity in chemical structure.

Flavonoids are derived from the amino acid l-phenylalanine *via* the general phenylpropanoid pathway, shown in Figure 1 (Winkel-Shirley, 2001). Chalcone synthase (CHS), the first committed enzyme of the flavonoid pathway, catalyzes the production of 2′,4,4′,6′-tetrahydroxychalcone (THC, **2**; Figure 1), which serves as a precursor for the other flavonoids (Austin and Noel, 2003). Aurones are directly derived from chalcones in limited plant species (Nakayama et al., 2000, 2001; Kaintz et al., 2014), while other flavonoids, including flavones, isoflavones, flavonols, and anthocyanidins, are derived after the conversion of chalcones to flavanones catalyzed by chalcone isomerase (CHI) (Winkel-Shirley, 2001). While the core flavonoid pathway is well conserved among seed plants, specific lineages develop specific flavonoid pathways to enhance fitness in particular environmental conditions. Enzymes involved in flavonoid biosynthesis (Figure 1) include polyketide synthases (e.g., CHS), 2-oxoglutarate-dependent dioxygenases [e.g., flavanone 3-hydroxylase (F3H, also termed FHT), anthocyanidin synthase (ANS; also termed leucoanthocyanidin dioxygenase, LDOX), flavonol synthase (FLS), flavone synthase I (FNSI)], short-chain dehydrogenases/reductases [e.g., dihydroflavanol 4-reductase (DFR)], aldo-keto reductases [e.g., chalcone reductase (CHR)], and cytochrome P450 monooxygenases [e.g., flavone synthase II (FNSII), flavonoid 3′-hydroxylase (F3′H), flavonoid 3′,5′-hydroxylase (F3′5′H), and 2-hydroxyisoflavanone synthase (IFS)]. These enzymes are hypothesized to have evolved from enzymes involved in primary metabolism (Weng and Noel, 2012; Moghe and Last, 2015). Cytochromes P450, shown with an asterisk in Figure 1, have been shown to be anchored to the cytoplasmic surface of the ER (Ralston and Yu, 2006), while most of the other enzymes are proposed to be soluble enzymes. A variety of regio-specific glycosyltransferases, acyltransferases, methyltransferases, and prenyltransferases acting on flavonoids have evolved in a lineage-specific manner to enhance the structural diversity of flavonoids (Ono et al., 2010; Sasaki and Nakayama, 2015).

It is generally accepted that the intracellular environments are of macromolecular crowding state (Fulton, 1982). Given our understanding of diffusion rates of small solutes and macromolecules in cells and organelles (Verkman, 2002), it is now recognized that cells and organelles are not simply bags of enzymes; rather, metabolic enzymes in the same pathway tend to be associated with each other in cellular environments, with each of these metabolic pathways confined to a specific region of the cell (microcompartmentalization of cellular metabolism) (Saks et al., 2008). The weakly bound, ordered complexes of enzymes involved in sequential metabolic pathways are referred to as “metabolons” (Ovadi and Srere, 2000; Srere, 2000; Ovadi and Saks, 2004; Jørgensen et al., 2005; Sweetlove and Fernie, 2013). The formation of a metabolon is believed to provide catalytic advantages *via* substrate channeling, including preventing the loss of intermediates by diffusion, reducing the transit time between active sites, protecting the chemically labile intermediates, circumventing unfavorable equilibria, and segregating the intermediates of competing reactions (Ovadi, 1991). The formation of metabolons is well defined in primary metabolic pathways of prokaryotic and eukaryotic cells, including glycolysis (Giege et al., 2003; Graham et al., 2007), the tricarboxylic acid cycle (Wu et al., 2015; Wu and Minteer, 2015), the Calvin-Benson cycle (Suss et al., 1993), and nucleotide synthesis (An et al., 2010). In plant specialized metabolism, metabolons formed during the biosynthesis of cyanogenic glycosides (Laursen et al., 2016) and lignins (Gou et al., 2018) in *Arabidopsis thaliana* (L.) Heynh. and other plant species have been studied in detail (Sweetlove and Fernie, 2013). In many cases, metabolon formation takes place on biological membranes or cytoskeletal elements *via* specific interactions of soluble enzymes with these cellular structures. However, because protein-protein interactions in metabolons are weak in most cases, it is difficult to isolate metabolons in their intact forms.

The concept of flavonoid metabolons was first proposed in 1974 to explain the efficiency of flavonoid synthesis in plant cells (Stafford, 1974). Subsequently, the association of flavonoid enzymes on biological membranes (e.g., the ER) and the formation of complexes were supported by several lines of experimental evidence (Hrazdina and Wagner, 1985a,b; Hrazdina et al., 1987; reviewed by Winkel, 2004). Since then, flavonoid metabolons have been assumed to form in diverse plant species; a model of flavonoid metabolon was proposed as a linear array of consecutive flavonoid enzymes along the ER (Hrazdina and Wagner, 1985a,b; Stafford, 1990). To date, specific protein-protein interactions in flavonoid metabolons have been studied in multiple plant species. Substrate channeling between DFR and leucoanthocyanidin 4-reductase (LAR) was predicted by computational studies, which also suggested the functional significance of metabolon formation during flavonoid synthesis (Diharce et al., 2016). Thus, elucidation of the structural organization of metabolons provides a basis for understanding how flavonoid structures are diversified, as well as how the temporal and spatial accumulations of flavonoids are regulated (Laursen et al., 2015). This review describes our current knowledge of specific protein-protein interactions identified in flavonoid metabolons and discusses their functional significance in flavonoid biosynthesis.

## Cytochromes P450 can be Components of Flavonoid Metabolons

It has been shown so far that soluble enzymes involved in plant specialized metabolism are associated on the cytoplasmic surface of ER to form metabolons, nucleated by ER-bound cytochromes P450. More than three decades ago, some of the soluble enzymes related to the general phenylpropanoid and flavonoid pathways, L-phenylalanine ammonia-lyase (PAL), CHS, and flavonoid glucosyltransferase, were found to be associated with the ER membrane in several plant species including *Hippeastrum* (amaryllis, order Asparagales) and *Fagopyrum esculentum* (order Caryophyllales) (Hrazdina and Wagner, 1985a,b; Hrazdina et al., 1987), suggesting the occurrence of ER-bound metabolons for the synthesis of phenylpropanoids and flavonoids. Meanwhile, the formation of metabolons during the syntheses of other classes of plant specialized metabolites, including cyanogenic glucosides and lignins, was shown to involve the anchoring of soluble enzymes by cytochromes P450 to specific domains of the ER membrane [reviewed by Ralston and Yu (2006)]. In 2004, in tobacco (*Nicotiana tabacum*, order Solanales), cinnamate 4-hydroxylase (C4H), a cytochrome P450 (CYP73A) that is involved in the general phenylpropanoid pathway (Figure 1), was found to be responsible for the weak association of soluble isozymes of PAL (PAL1 and PAL2) with ER membranes (Figure 1), using a combination of biochemical and fluorescence microscopic methods (Achnine et al., 2004).

**Figure 2:**
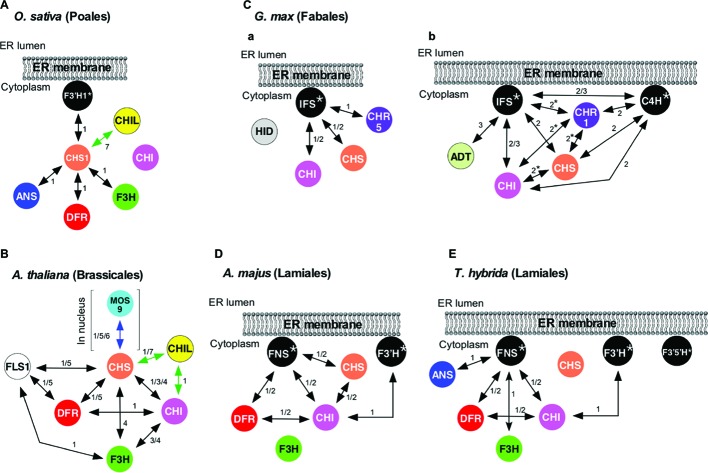
Protein-protein interactions among flavonoid enzymes. Black, double-headed arrows denote interactions between flavonoid enzymes (circles; see Figure 1 for enzyme abbreviations), with numerals indicating methods used for detection of protein-protein interactions: 1, Y2H and split-ubiquitin Y2H; 2, BiFC; 3, IP; 4, AC; 5, FRET; 6, SPR; and 7, LuCIA. Green and blue double-headed arrows denote interactions between flavonoid enzymes and proteins with no enzymatic activity. **(A)** Interactions among flavonoid enzymes and proteins in *O. sativa*. **(B)** Interactions among flavonoid enzymes and proteins in *A. thaliana*. FLS1 and DFR interact with CHS in a mutually exclusive manner *in planta*. **(C)** Interactions among flavonoid enzymes in *G. max* L. (Merr.) proposed by (**a**) Waki et al. (2016) and Mameda et al. (2018) and (**b**) Dastmalchi et al. (2016). Interactions with asterisks were reported to be absent or very weak by other groups. **(D)** Interactions among flavonoid enzymes in *A. majus*. **(E)** Interactions among flavonoid enzymes in *T. hybrida*. Interactions shown here do not necessarily take place simultaneously. Also, because temporal and spatial expression patterns of enzymes and proteins involved in flavonoid biosynthesis may vary, metabolon components could be different in cells from different organ/tissues and at different growth stages.

Formation of a flavonoid metabolon on cytochrome P450 was first demonstrated in 2008 in rice (*Oryza sativa* L.; order Poales, a monocot) that accumulates flavones, flavonols, proanthocyanidins (oligomeric flavan-3-ols), and anthocyanins (Shih et al., 2008). In this plant, an isozyme of flavonoid 3′-hydroxylase (F3′H1), a cytochrome P450 (CYP75B) catalyzing the 3′-hydroxylation of the B-ring of flavonoids (Figure 1), was shown to bind to CHS1 (a CHS isozyme) (Figure 2A) by yeast two-hybrid (Y2H) assays (Shih et al., 2008). The series of binary interaction assays showed that CHS1 also interacts with F3H, DFR, and ANS1 (an isozyme of rice ANS), but not with CHI (Figure 2A). Interactions among CHI, F3H, F3′H1, DFR, and ANS1 were not detected. It was proposed that in rice, CHS could serve as a common platform for a flavonoid metabolon, which might be anchored to the cytoplasmic surface of the ER *via* F3′H1. In 2016, two groups independently published evidence supporting the association of soybean flavonoid enzymes in metabolons tethered to the ER-bound cytochromes P450 IFS (CYP93C) and C4H (Figures 2Ca,Cb; Dastmalchi et al., 2016; Waki et al., 2016; Mameda et al., 2018). Additionally, physical interactions among flavonoid enzymes in snapdragon (*Antirrhinum majus* L.) and torenia (*Torenia hybrida*) were clarified, in which FNSII (CYP93B1, Figures 2D,E) was found to be a component of flavonoid metabolons (Fujino et al., 2018) (see below).

## Flavonoid Diversity and Flavonoid Metabolons

During the past two decades, physical interaction partnerships of flavonoid enzymes and related proteins have been studied in multiple phylogenetically distinct plants, including rice, *A. thaliana*, soybean, snapdragon, and hops (*Humulus lupulus* L. var. *lupulus*), each of which belongs to different orders of plants and accumulates different classes of flavonoids (see below). The data suggest that production of specific flavonoids in these plants is attained *via* spatially and temporally dependent interactions between specific proteins during plant growth and stress responses. These data are discussed in more detail below.

## Flavonoid Metabolons in *Arabidopsis*

*A. thaliana* (order Brassicales), in which 54 flavonoid species have been identified to date, primarily accumulates flavonols and proanthocyanidins and also produces anthocyanins under stress conditions (Saito et al., 2013). Studies of *A. thaliana* flavonoid enzymes provide one of the best-characterized flavonoid metabolons with respect to protein-protein interactions. Direct protein-protein interactions among soluble flavonoid enzymes in *A. thaliana* have been studied by Y2H assays, affinity chromatography (AC), immunoprecipitation (IP), and physicochemical methods: Förster resonance energy transfer (FRET) detected by fluorescence lifetime imaging microscopy (FLIM) and surface plasmon resonance refractometry (SPR) (Burbulis and Winkel-Shirley, 1999; Owens et al., 2008; Crosby et al., 2011; Watkinson et al., 2018). In this plant, interactions between the following enzyme pairs have been identified (followed by methods in parentheses): CHS-CHI (Y2H, AC, and IP), CHS-F3H (AC), CHS-DFR (Y2H and FRET), CHS-isozyme of FLS (FLS1) (Y2H and FRET), CHI-DFR (Y2H), CHI-F3H (AC and IP), FLS1-DFR (Y2H and FRET), and FLS1-F3H (Y2H) (Figure 2B, black arrows). Interactions of catalytically inactive paralogs of FLS with CHS and DFR were also found *via* Y2H (Owens et al., 2008). These binary interactions suggested a flavonoid metabolon model (Figure 2B) with CHS as the hub. Moreover, this model features a globular association, rather than a linear array, of flavonoid enzymes in the metabolons. FRET-FLIM analyses revealed that FLS1 and DFR, the key enzymes of branch pathways (Figure 1), interact with CHS in a mutually exclusive manner *in planta* (Crosby et al., 2011). This provides a possible *in planta* mechanism for regulating metabolic flux by changing physical interactors with CHS, which is pivotal in the pathway.

It has been shown that not only flavonoid enzymes but also a protein with no catalytic activity can be a component of the flavonoid metabolon in *A. thaliana*. Phylogenetic analyses suggest that CHI enzymes have evolved from a non-catalytic ancestor related to fatty acid-binding proteins (FAPs) and land plant-specific CHI-like proteins (CHILs) with no catalytic activity (Ngaki et al., 2012; Kaltenbach et al., 2018). Thus, CHIs, FAPs, and CHILs, all of which are soluble proteins, constitute a larger structurally related family, the CHI-fold family, in which CHIs correspond to types I and II, and FAPs and CHILs, respectively, correspond to types III and IV within the family. In 2014, CHILs were shown to serve as enhancers of flavonoid production (EFPs), as loss-of-function mutations and suppression in morning glory (*Ipomoea nil*) and torenia, respectively, resulted in a significant diminution of flavonoid contents (Morita et al., 2014). CHIL is also produced by *A. thaliana*. Y2H analyses indicated that in *A. thaliana*, CHIL binds to CHI (Figure 2B, green arrow), suggesting that CHIL is a component of the flavonoid metabolon (Jiang et al., 2015). Recently, CHIL of *A. thaliana* (AtCHIL) was also shown to physically interact with CHS of the same plant species (AtCHS) (Figure 2B, green arrow) by Y2H and luciferase-complementation imaging assays (LuCIA) (Ban et al., 2018). The coexpression of *AtCHIL* with *AtCHS* in yeast *Saccharomyces cerevisiae* resulted in a 1.8-fold enhancement of AtCHS-catalyzed production of THC. The interactions of CHIL with CHI and CHS might be related to the observed role of CHIL as an EFP. It must be mentioned that the binding of CHIL to CHS has also been observed in the flavonoid systems of hops, rice (Figure 2A), *Selaginella moellendorffii* (a lycophyte), and *Physcomitrella patens* (a bryophyte), as assayed by LuCIA (Ban et al., 2018). The coexpression of CHILs with CHS of these plants in *S. cerevisiae* also enhanced the CHS-catalyzed production of THC, suggesting the conservation of the EFP role of CHIL proteins throughout land plants.

The dynamism and versatility of CHS-mediated protein-protein interactions likely take place in organ- and organelle-specific manners in *A. thaliana*. Immunofluorescence and immunoelectron microscopic analyses showed that CHS and CHI co-localize at the ER and tonoplasts in epidermal and cortex cells of *A. thaliana* roots (Saslowsky and Winkel-Shirley, 2001). This observation suggests that a subset of CHS and CHI enzymes in root cells might not be assembled into metabolons that are mentioned above. As both of these enzymes are soluble, these data suggest that one or more other proteins function in recruiting these enzymes to membranes, although this protein remains to be identified. It remains to be determined whether CHS and CHI interact with cytochromes P450 in *A. thaliana*. In this context, in *A. thaliana* root cells, F3′H is unlikely to be involved in recruiting CHS and CHI to the ER, as suggested by the results of immunolocalization in the *A. thaliana* F3′H mutant *tt7*(88) (Saslowsky and Winkel-Shirley, 2001).

CHS and CHI have also been shown in the nucleus of *A. thaliana* by multiple immunolocalization methods (Saslowsky et al., 2005). CHS of *A. thaliana*, a dimeric enzyme, possesses sequences resembling a nuclear localization signal, which is located on the surface opposite from the dimerization interface. This signal could direct CHS and associated enzymes into the nucleus. Moreover, immunoblotting of nuclear CHI suggested post-translational modifications that also might be responsible for the nuclear localization of the enzyme. Interestingly, CHS was recently found to interact with MOS9 (a nuclear protein associated with epigenetic control of *R* genes that mediate effector-triggered immunity) as analyzed by Y2H, SPR, and FRET, with a *K*_d_ of 210 nM (Figure 2B, blue arrow) (Watkinson et al., 2018). Addressing this finding further may uncover additional mechanisms for controlling flavonoid pathways, as well as linking them to defense mechanisms and other physiological functions.

## The Soybean Isoflavonoid Metabolon

**Figure 3:**
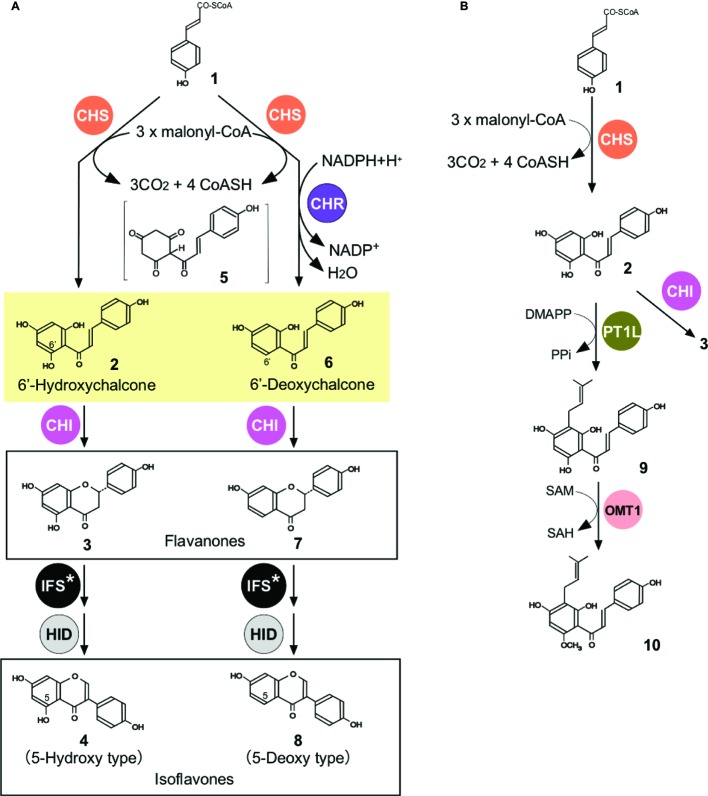
Biosynthesis of flavonoids in soybean **(A)** and hops **(B). (A)** Biosyntheses of 5-hydroxy- and 5-deoxyisoflavonoids in soybean. CHR, chalcone reductase. See Figure 1 for abbreviations for other enzymes. **(B)** Biosyntheses of prenylated flavonoids in hops. PT, aromatic prenyltransferase; OMT, *O*-methyltransferase; DMAPP, dimethylallyl diphosphate; PPi, pyrophosphate; SAM, *S*-adenosyl-L-methionine; SAH, *S*-adenosyl-L-homocysteine. Flavonoids and related metabolites are *p*-coumaroyl-CoA (**1**), THC (**2**), naringenin (**3**), genistein (**4**), *p*-coumaroylcyclohexantrione (**5**), isoliquiritigenin (**6**), liquiritigenin (**7**), daidzein (**8**), demethylxanthohumol (**9**), and xanthohumol (**10**).

The soybean (order Fabales) produces isoflavones, which are a class of flavonoids with a 3-phenylchromone structure and distributed almost exclusively in legumes (Aoki et al., 2000). Isoflavones play important roles in symbiotic plant-microbe interactions and defensive mechanisms against pathogen infection in soybean (Barz and Welle, 1992). Moreover, soybean isoflavones show a variety of bioactivities that are beneficial to human health (Wiseman, 2006). The soybean produces two distinct types of isoflavonoids: 5-deoxyisoflavonoids (daidzein and its conjugates) and 5-hydroxyisoflavonoids (genistein and its conjugates) (**4** and **8**, respectively, Figure 3A). In unstressed soybean plants (cv. Enrei), 5-deoxyisoflavonoids accumulate in the roots (93% mol/mol of total root isoflavonoids) and seeds (60% mol/mol of the total seed isoflavonoids) (Mameda et al., 2018).

### Characterization of Isoflavonoid Metabolon

Protein-protein interaction analyses of soybean isoflavonoid enzymes suggested that biosynthesis of isoflavones takes place *via* the formation of a metabolon on cytochromes P450. Specifically, the analysis using split-ubiquitin Y2H and bimolecular fluorescence complementation (BiFC) assay systems revealed that each enzyme located upstream of the isoflavonoid pathway (CHS, CHI, and GmCHR5 (an isozyme of soybean CHR); Figures 1, 3A) interacts with isozymes of IFS (CYP93C) to form a metabolon (Figure 2Ca; Waki et al., 2016; Mameda et al., 2018). It has been proposed that C4H also serves as a nucleus for the metabolon formation as analyzed *via* BiFC and IP (Figure 2Cb; Dastmalchi et al., 2016). Moreover, arogenate dehydratase (ADT), a shikimate pathway enzyme that has primarily been reported to be a plastidial enzyme, was reported to interact with IFS on the basis of IP (Figure 2Cb; Dastmalchi et al., 2016). The fluorescence localizations observed during these BiFC analyses were consistent with P450-mediated interactions taking place at the ER (Waki et al., 2016). As the activities of IFS and C4H are indispensable for the formation of isoflavones, these cytochromes P450 are considered to play both catalytic and structural roles in the metabolon.

The affinity of the soybean isoflavonoid enzymes for IFS isozymes varies among paralogs. Isoflavonoid enzymes shown in Figure 2C are encoded by multiple genes in soybean. For example, there are at least nine paralogs (GmCHSs) encoding CHS (Schmutz et al., 2010; Shimomura et al., 2015), 12 encoding CHI (Shimada et al., 2003; Ralston et al., 2005), and two encoding IFS (Cheng et al., 2008). For each enzyme, different isozymes exert different physiological functions (Shimizu et al., 1999; Tuteja et al., 2004; Livingstone et al., 2010). The analysis using split-ubiquitin Y2H system suggested GmCHS1 has a higher affinity for GmIFS1 than for GmCHS7 (Waki et al., 2016), and GmCHR5 binds to GmIFS isozymes but other GmCHR isozymes cannot (see below for details) (Mameda et al., 2018). These observations could be related to differential regulation and physiological roles of each enzyme paralog.

### An Implication for Functional Significance of Protein-Protein Interactions During 5-Deoxyisoflavonoid Biosynthesis

Although 5-deoxyisoflavonoids accumulate in the roots and seeds of unstressed plants in a high ratio (Mameda et al., 2018), its mechanistic details remained unknown. During the course of 5-deoxyisoflavonoid biosynthesis, isoliquiritigenin (Figure 3A, **6**) (a 6′-deoxychalcone) is produced *via* a CHS-catalyzed reaction coupled to CHR catalysis (Bomati et al., 2005). The soybean genome encodes 11 CHR paralogs (Mameda et al., 2018), among which only GmCHR1 had been characterized enzymatically (Welle and Grisebach, 1988; Welle et al., 1991). Although CHR has been referred to as chalcone reductase, it does not actually act on THC (**2**, Figure 3A) but instead on one of the diffusible intermediates of the CHS-catalyzed reaction, most likely *p*-coumaroylcyclohexantrione (**5**, Figure 3A), which is highly unstable and is rapidly aromatized to produce THC in an aqueous system (Bomati et al., 2005). THC and isoliquiritigenin then undergo the reactions catalyzed by CHI, IFS, and 2-hydroxyisoflavanone dehydratase (HID) to produce genistein (**4**) and daidzein (**8**), respectively (Figure 3A; Mameda et al., 2018). The amount of the CHR product isoliquiritigenin does not generally exceed 25% (mol/mol) of the total CHS products (isoliquiritigenin, THC, and naringenin) during the combined action of dilute CHS and GmCHR1 (0.05 μM each) *in vitro*. These low product ratios for CHR catalysis during *in vitro* assays could arise from the fact that only a small fraction (<25%) of **5** produced during CHS catalysis is transferred to the active site of GmCHR1 while the majority (>75%) escapes and diffuses to the aqueous system to give rise to THC.

To establish a high 5-deoxyisoflavonoid ratio in the cells of soybean roots and seeds (i.e., a high product ratio for CHR catalysis), **5** has to be immediately transferred, prior to aromatization, from the active site of CHS to that of CHR. One possible mechanism for achieving this would be binding of CHR to CHS, facilitating the channeling of **5** between them. However, the crystal structure of CHR suggested that direct association of the active sites of CHR and CHS is impossible and that passive diffusion may be the only way to transfer **5** from CHS to CHR (Bomati et al., 2005). In fact, Y2H assays showed that GmCHR1 (the only GmCHR paralog whose catalytic activity was confirmed) was unable to interact with any of the GmCHS isozymes (Waki et al., 2016; Mameda et al., 2018). Alternatively, a shorter distance or transit time for **5** between the two enzymes could be achieved in a metabolon and located very close to each other. Because CHS isozymes have been shown to interact with IFS isozymes (GmIFS) (Figure 2Ca; Dastmalchi et al., 2016; Waki et al., 2016), the involvement of GmCHR1 in the isoflavonoid metabolon was examined. However, GmCHR1 was not found to interact with any of the enzymes examined including IFS isozymes (Waki et al., 2016; Mameda et al., 2018). Moreover, the product ratio for CHR catalysis did not exceed 50% even when high concentrations of GmCHR1 and CHS were used in *in vitro* enzyme assays (Oguro et al., 2004; Mameda et al., 2018). Therefore, the involvement of GmCHR1 in the observed high proportion of 5-deoxyisoflavonoids in the roots and seeds of unstressed plants was unlikely.

Thus, 11 GmCHR paralogs were comprehensively analyzed for their possible involvement in biosynthesis of 5-deoxyisoflavonoids in the roots and seeds of unstressed plants, and the data obtained strongly suggested the involvement of a previously unappreciated soybean CHR, GmCHR5 (Figure 2Ca). Specifically, among the GmCHR paralogs examined, the expression patterns of *GmCHR5* were the most consistent with the observed patterns of the accumulation of daidzein conjugates in the roots and the seeds of unstressed plants. When interactions of these GmCHR isozymes with soybean isoflavonoid enzymes (Figure 3A) were analyzed by split-ubiquitin Y2H assays, GmCHR5 uniquely interacted with IFS isozymes (Mameda et al., 2018). Moreover, *in vitro* assay results suggested that the product ratio for CHR catalysis depended on the GmCHR5 concentration, with higher concentrations resulting in higher ratios (approaching 90%) (Mameda et al., 2018). Thus, the results of enzyme assays, transcription analyses, and protein-protein interaction assays were all consistent with the fact that GmCHR5, but not other CHR isozymes, is the key player in the accumulation of 5-deoxyisoflavonoids in the roots and seeds of unstressed plants. It would be highly likely that the interactions of CHS and GmCHR5 with IFS could allow the microcompartmentalization of the metabolic process, resulting in a product ratio for CHR catalysis high enough for the dominated accumulation of 5-deoxyisoflavonoids in the roots and seeds of unstressed plants. This illustrates the previously proposed functional significance of metabolon formation, i.e., preventing the loss of intermediates by diffusion and reducing the transit time between active sites. This also supports the hypothesis that specific spatial distributions of a flavonoid can be attained by inclusion of a specific isozyme in a flavonoid metabolon in a spatially specific manner.

### Functional Differentiation of GmCHR Isozymes in the Soybean

GmCHR1 and GmCHR6 are unable to interact with any of the isoflavonoid enzymes shown in Figure 3A (Mameda et al., 2018). However, this does not necessarily rule out their involvement in 5′-deoxyisoflavonoid biosynthesis but is rather consistent with functional differentiation of GmCHR isozymes in the soybean. Previously, expression of *GmCHR1, GmCHR5*, and *GmCHR6* was shown to be induced upon microbial infection (Sepiol et al., 2017). Moreover, *GmCHR6* is located near a quantitative trait locus region linked to resistance to a pathogenic oomycete (Sepiol et al., 2017). In soybean, the production of both types (5-deoxy- and 5-hydroxy-) of isoflavonoids is induced by microbial pathogens. The production of both types of isoflavonoids, rather than the exclusive production of 5′-deoxy type, would be needed to fully implement relevant soybean defense mechanisms. The induced production of GmCHR1 and GmCHR6, showing the maximum product ratio for CHR catalysis of 50%, make it possible to accumulate high levels of both types of isoflavonoids in infected plants. Thus, it would be likely that GmCHR5 plays a key role in the exclusive accumulation of 5-deoxyisoflavonoids in the roots and seeds of unstressed plants while GmCHR1 and GmCHR6 play key roles in the induced defense mechanisms against microbial pathogens.

## Flavonoid Metabolons in the Order Lamiales

Snapdragon and torenia are flowering ornamentals in which colorful petals are the most eye-catching trait. The petal colors in these lamiales plants are mainly provided by flavonoids, which represent different flavonoid classes from those mainly found in *A. thaliana* and soybean. The petal colors of snapdragon—magenta, orange, red, pink, yellow, cream, and white—are produced by a combination of anthocyanins (orange, pink, red, and reddish purple), aurones (yellow), and flavones (co-pigments) (Ono and Nakayama, 2007). Torenia accumulates anthocyanins and flavones in its flower petals, which are responsible for the bluish purple and pink colors (Ueyama et al., 2002).

In 2018, physical interactions among flavonoid enzymes in snapdragon and torenia were clarified, illustrating the formation of flavonoid metabolons responsible for flower coloration (Fujino et al., 2018). Binary interactions found in split-ubiquitin Y2H and BiFC assays were: FNSII-CHS, FNSII-CHI, FNSII-DFR, CHS-CHI, CHI-DFR, and F3′H-CHI in snapdragon; and FNSII-CHI, FNSII-F3H, FNSII-DFR, FNSII-ANS, CHI-DFR, and F3′H-CHI in torenia (Figures 2D,E). Split-ubiquitin Y2H assays also suggested that binding of CHI and DFR to FNSII is not exclusive in snapdragon.

Interestingly, enzymes involved in the late stage of anthocyanin biosynthesis (DFR in snapdragon; DFR, F3H, and ANS in torenia) were found to interact with FNSII (cytochrome P450 CYP93B1) (Figures 1, 2D,E; Fujino et al., 2018). The activity of FNSII is not needed for anthocyanin biosynthesis, suggesting that FNSII could function as a scaffold for anthocyanin biosynthesis. Although further studies are needed to test this hypothesis, several findings are consistent with FNSII as an important component of the metabolon for anthocyanin biosynthesis. Previously, attempts were made to engineer torenia flowers showing a deeper petal color using metabolic engineering (Ueyama et al., 2002). To achieve this, *FNSII* was co-suppressed in blue-violet torenia flowers to diminish FNSII activity. As anthocyanin synthesis competes with flavone synthesis for flavanones as the shared precursors (Figure 1), this was predicted to favor anthocyanin production at the expense of flavone formation (see Figure 1). This strategy was inspired by the observations in black dahlia (*Dahlia variabilis*, order Asterales) accumulating large amounts of anthocyanins, in which FNSII production is suppressed by endogenous posttranscriptional gene silencing (Thill et al., 2012; Deguchi et al., 2013). In the black dahlia, suppression of *FNSII* increased production of anthocyanins while flavone production was decreased. Metabolic engineering of torenia showed that the co-suppression of *FNSII* diminished flavone and increased flavanone levels in petals, as expected (Ueyama et al., 2002). However, anthocyanin levels in the petals of the *FNSII*-suppressed torenia decreased considerably, producing a paler flower. The reason for this result was unknown, but this observation can now be explained by FNSII acting as a component of the metabolon related to anthocyanin production.

Interestingly, in the anthocyanin-accumulating snapdragon petals, flavones were accumulated first, followed by anthocyanins, and finally aurones (Toki, 1988; Fujino et al., 2018). This sequence of flavonoid accumulation is consistent with the transcriptional patterns of snapdragon flavonoid enzyme genes during flower development (Fujino et al., 2018). Thus, on the basis of interactions (Figure 2D) and temporal gene expression patterns of flavonoid enzymes in red snapdragon petal cells, a model of the flower stage-dependent formation of the flavonoid metabolon has been proposed (Fujino et al., 2018). In this model, CHS, CHI, and FNSII are expressed and form a flavone metabolon on the ER surface at the beginning of the flower development. Halfway through flower development, F3H and DFR are expressed to form an anthocyanin metabolon by using the preexisting flavone metabolon as a scaffold.

The similarity of interaction partnerships in the flavonoid metabolons of snapdragon and torenia (Figures 2D,E) is consistent with the close phylogenetic relationship of these plants. Collectively with the fact that the *A. thaliana* genome lacks *IFS* and *FNSII* genes, interactions in flavonoid metabolons may differ between plant species while those of closely related plant species are more similar to each other (Figure 2). This is consistent with the observed structural diversity of flavonoids in plants and the fact that each plant lineage produces structurally distinct flavonoids in a lineage-specific manner.

## Protein-Protein Interactions of Flavonoid Enzymes and Proteins in Hops

Hops uniquely accumulate the prenylated flavonoids xanthohumol (3′-prenyl-6′-*O*-methyl-THC) (**10**, Figure 3B) and demethylxanthohumol (3′-prenyl-THC) (**9**) in the glandular trichomes (lupulin glands) of female cones, a key ingredient in beer brewing (Stevens and Page, 2004). Recent studies of the synthesis of these prenylated flavonoids provide examples of the involvement of non-catalytic CHI-fold proteins in flavonoid metabolons as specialized auxiliary proteins (Ban et al., 2018).

In hops, the soluble, trichome-specific isozyme of CHS (CHS_H1) is involved in the biosynthesis of prenylchalcones and catalyzes the production of THC (**2**, Figure 3B); THC is then prenylated by the membrane-bound, aromatic prenyltransferase PT1L, then 6′-*O*-methylated by the soluble *O*-methyltransferase OMT1 to produce xanthohumol (**10**, Figure 3B; Ban et al., 2018). Recent studies have shown that non-catalytic members of the CHI-fold protein family, CHIL1 (a type-III, FAP-related protein) and CHIL2 (a type-IV, EFP-related protein), are involved in the syntheses of prenylated flavonoids in hops. CHIL2 was found to interact with CHS_H1 and PT1L by Y2H, LuCIA, and IP assays (Ban et al., 2018). As PT1L is a membrane-bound enzyme with eight predicted transmembrane domains and proposed to localize in trichome plastids (Li et al., 2015), these results suggest a membrane-anchored metabolon for xanthohumol biosynthesis. *In vitro* enzymatic assays showed that CHIL2 slightly enhances the catalytic efficiencies of CHS_H1 and PT1L. Specifically, the binding of CHIL2 to CHS_H1 results in a 6–18-fold increase in *k*_cat_ and 5.5–6.0-fold increase in *K*_m_ for *p*-coumaroyl-CoA (**1**, Figure 3B) and malonyl-CoA, with up to 2.9-fold increase in *k*_cat_/*K*_m_ values; whereas, the binding of CHIL2 to PT1L results in a slight increase in *V*_max_ and slight decrease in *K*_m_ for these substrates, with up to a 2.2-fold increase in *V*_max_/*K*_m_ values. *S. cerevisiae* was engineered for the production of demethylxanthohumol. The engineered yeast co-expressing CHIL2 and CHS_H1 with PT1L produced greater amounts of demethylxanthohumol than those expressing CHS alone, consistent with CHIL2 functioning as an EFP *in vivo*. Thus, specific binding of CHIL2 to CHS enhances the rate of CHS-catalyzed entry from the general phenylpropanoid pathway to the flavonoid pathway (Figure 1) to potentiate flavonoid production. Unlike CHIL2, CHIL1 did not interact with CHIL2, PT1L, CHS_H1, *p*-coumaroyl-CoA ligase (see Figure 1), or OMT, as found by multiple methods (Ban et al., 2018). Binding assays and computational docking studies suggested that CHIL1 binds to demethylxanthohumol and THC to stabilize their ring-opening conformations, circumventing isomerization of THC to naringenin flavanone (Ban et al., 2018). This role of CHIL1 is consistent with the high accumulation of xanthohumol and demethylxanthohumol in hop glandular trichomes, in which almost no THC and naringenin were detected. PT1L is also involved in bitter acid biosynthesis in this plant and physically interacts with another membrane-bound, plastidial, aromatic prenyltransferase, PT2, to form a metabolon that catalyzes the prenylations in the β-bitter acid pathway. In this pathway, PT1L catalyzes the first prenylation and PT2 catalyzes the subsequent two prenylations (Li et al., 2015; Ban et al., 2018). Thus, PT1L might serve as a key scaffold for the biosynthesis of both terpenophenolics (bitter acids and prenylated flavonoids) in hop glandular trichomes.

## Conclusion and Future Perspectives

Interactions between enzymes and proteins in the flavonoid metabolons clearly vary between plant species. This is consistent with the species-dependent structural diversity of flavonoids and points to a role for differential metabolon formation in producing different structures of flavonoids in a plant species-specific manner. Although specific protein-protein interactions in some flavonoid metabolons have been identified, it is still difficult to grasp the larger picture of flavonoid metabolons and understand how enzymes and proteins dynamically form metabolons to regulate flavonoid biosynthesis. Specifically, the flavonoid metabolon models proposed to date are primarily based on the results of binary interaction analyses and do not describe how three or more enzymes and/or scaffolding proteins are simultaneously and cooperatively associated. Moreover, the protein-protein interactions in flavonoid metabolons remain to be investigated at the atomic level, and their functional significance is yet to be addressed in most important plant taxa. Finally, it is likely that the interactions underlying metabolon formation have evolved to implement higher orders of metabolic functions in the cell, allowing for the structural diversification of flavonoids. Taking full advantage of these important phenomena in synthetic biology has the potential to enhance the efficiency of production of many useful flavonoids in heterologous systems.

## Author Contributions

TN mainly wrote the manuscript and was responsible for the general opinions stated in the manuscript. All authors reviewed and agreed with the final version of the submitted manuscript.

### Conflict of Interest Statement

The authors declare that the research was conducted in the absence of any commercial or financial relationships that could be construed as a potential conflict of interest.
